# Effects of Acute and Chronic Restraint Stress on Reinstatement of Extinguished Methamphetamine-induced Conditioned Place Preference in Rats

**DOI:** 10.29252/nirp.bcn.9.3.157

**Published:** 2018

**Authors:** Zahra Taslimi, Alireza Komaki, Abbas Haghparast, Abdolrahman Sarihi

**Affiliations:** 1. Neurophysiology Research Center, Hamadan University of Medical Sciences, Hamadan, Iran.; 2. Neuroscience Research Center, School of Medicine, Shahid Beheshti University of Medical Sciences, Tehran, Iran.

**Keywords:** Reward, Stress, Methamphetamine (METH), Reinstatement, Conditioned place preference

## Abstract

**Introduction:**

Methamphetamine (METH) is a neurotoxic psychostimulant with highly addictive potential that leads to compulsive drug use and vulnerability to relapse. Environmental cues, such as drug exposure, peer influence, and social stress, are the powerful triggers of drug relapse. In this study, we tried to find out the effect of acute and chronic restraint stress on reinstatement of extinguished METH-induced Conditioned Place Preference (CPP) in rats.

**Methods:**

Subcutaneous (SC) administration of METH (0.125, 0.25, 0.5, 1, 2 and 4 mg/kg) could induce CPP and it was found that METH with the dose of 0.5 mg/kg was more potent than other doses. In extinction phase, rats were put in the CPP box for 30 min per day for 8 consecutive days. After extinction, animals were exposed to restraint stress (3-h period, as an acute stress) 60 min before subcutaneous administration of ineffective dose of METH (0.125 mg/kg) in order to reinstate the extinguished METH-induced CPP. For induction of the chronic stress during extinction phase, the animals were exposed to the restraint stress for one hour per day.

**Results:**

The results showed that the effective dose of METH to induce CPP was 0.5 mg/kg. Based on the results, physical stress (restraint stress) whether acute and chronic, can significantly induce reinstatement of METH-induced CPP (P˂0.001) in extinguished animals.

**Conclusion:**

Additionally, the chronic restraint stress could reduce duration of extinction (maintenance) of METH-induced CPP. It seems that exposure to the stress induces the relapse in abstinent amphetamine, but acute and chronic situation have a different reaction.

## Highlights

- Acute restraint stress potentiates the reinstatement of extinguished methamphetamine-induced CPP.
- Chronic restraint stress potentiates the reinstatement of extinguished methamphetamine-induced CPP.
- Chronic restraint stress reduces duration of extinction phase in methamphetamine-treated rats.

## Plain Language Summary

Methamphetamine (METH) is a global epidemic drug of abuse. It is a potent central nervous system (CNS) stimulant which is mainly used as a recreational drug; however, it is highly addictive with vulnerability to relapse (return to drug use). Many environmental factors like stress lead to METH reuse. In this study, we tried to find out the effect of acute and chronic restraint stress on METH reuse. Several doses of METH subcutaneously injected in rats. Effective dose of METH for conditioning was selected (0.5 mg/kg). Animals were put in a three-compartment apparatus and conditioned by METH in one compartment during 3 day. Conditioning was extinguished by putting animals in conditioned compartment without METH injection which takes 8 days. After that, the animal received METH ineffective dose usually (the dose of METH in the first day of exposure that could not induce place conditioning) returns to conditional conditions. To investigate the effects of acute stress, the animals were exposed to restraint stress (3-h period, as an acute stress) 60 min before METH (0.125 mg/kg) injection. In another experiment, the animals were exposed to the restraint stress for a 1-h period/day during the days which they were losing their METH place conditioning. Finally, these groups of animal received an ineffective dose of METH (0.125 mg/kg). The results showed that acute and chronic restraint stress significantly impact the effects of METH rewards. However, stress could affect the ineffective doses of METH, while in stress-free conditions, it could not. Additionally, the chronic stress could reduce duration of conditional losing (Less than 8 days). Therefore, it seems that exposer to stress facilitates the return to drugs such as METH, but acute and chronic stress has different functional roles.

## 1. Introduction

Methamphetamine (METH) is a psychostimulant that reinforces behavioral responses and persuades compulsive medicine use and vulnerably to relapse (National Institute on Drug Abuse, 2010). Though the precise neurobiological mechanisms underlying METH addicting behavior remain unknown, while rewarding effect of the drug plays a critical role. In addition, relapse is the most difficult challenge in the treatment of addiction, and its neurobiological mechanisms are still unclear (Sulzer, Sonders, Poulsen, & Galli, 2005). The relationship between stress and drugs has been successfully patterned in rodents and various acute and chronic stressors trigger drug-seeking behavior in them (Conrad et al., 2010; Faravelli et al., 2012). Furthermore, it has been proved that preference for the drug-paired environment can be reinstated by drug priming injections or stressors (Conrad et al., 2010; Sinha, 2008).

Specialized medical researchers have indicated that stress is not only a risk factor in the development of habit but also an urge trigger to drug maltreatment. However, the mechanisms of stress-induced drug relapse are still a matter of debate (De Giovanni, Guzman, Virgolini, & Cancela, 2016). In fact, stressful activities modify the experience of brain areas active in the rewarding effects of psychostimulants (Belujon & Grace, 2011). Also, it has been suggested that environmental stressors produce long-term changes in the function of brain reward pathways in the same way as drugs of abuse do (Quadros & Miczek, 2009).

Exposure to stress increases drug-seeking behavior and the risk of addictive drug use in human and animal models by the mechanisms that are not completely understood yet (Karimi, Attarzadeh-Yazdi, Yazdi-Ravandi, Hesam, Azizi, Razavi, et al., 2014). Immobilization stress is a kind of psychological stress that produces two major disruptions described in the literary works, decrease in food intake (Marti, Marti, & Armario, 1994) and creation of anxiety (Vyas, Mitra, Shankaranarayana Rao, & Chattarji, 2002; Sotomayor-Zarate et al., 2015). Restraint stress has been used to stimulate reinstatement of extinguished choice in CPP (conditioned place preference) trained animals for different drugs such as METH (Han, Du, Fu, Wang, Song, Wu, et al., 2014), nicotine (Leao, Cruz, & Planeta, 2009) and cocaine (Briand & Blendy, 2013).

Acute stress is sudden and of short duration. This stress results from specific events or situations that involve novelty, unpredictability, a threat to the life, and live with a poor sense of control. While chronic stress is a long-term stuff and unabated stress, resulting from repeated direct exposure to situations that lead to the discharge of stress hormones (Koob, 2008). Stress increases drug-seeking behavior and the risk of addictive drug whose mechanisms are not clearly understood yet. Consequently, in this study, we attempted to examine the effects of acute and chronic restraint stress on the reinstatement of extinguished METH CPP in rats.

## 2. Methods

### 2.1. Study animals

The study animals were housed in groups of four in a 12/12 h light/dark cycle (light on between 7:00 AM and 7: 00 PM) with free access to food and water (Parvishan, Taslimi, Ebrahimzadeh, & Haghparast, 2011). Adult male albino Wistar rats (Pasteur Company, Tehran, Iran) weighing 200–280 g were used in these experiments. The animals were randomly assigned to control and treatment plan groups. Each animal was used only once. Rats got familiar with their new environment prior to starting experimental process. All tests were executed according to the guide for the care and use of laboratory animals (National Institutes of Health Newsletter No. 80-23, revised 1996). The study was approved by the Research and Ethics Committee of Hamadan University of Medical Sciences, Hamadan, Iran.

### 2.2. Drugs

In the present study, METH (Purity ˃98%, donated by the Iran’s Drug Control Headquarters) dissolved in sterile saline was used.

### 2.3. Apparatus

#### 2.3.1. Conditioning place preference paradigm

A three-compartment CPP apparatus (30 cm×30 cm×40 cm) was used in these experiments (Haghparast, Taslimi, Ramin, Azizi, Khodagholi, & Hassanpour-Ezatti, 2011). Place conditioning was conducted using an un-biased procedure. The apparatus was made of Plexiglas which was divided into three compartments, two equal size with different textured compartments and one smaller size as a null compartment by means of a removable wall, but distinguishable by texture. To provide the tactile difference between the compartments, one of the compartments had a smooth floor while the other compartment had a net-like floor. Two preference compartments were differently striped black and white on their walls. The null compartment was a red tunnel (30 cm×15 cm×40 cm) connecting the two preference compartments. In this apparatus, rats showed no consistent preference for either of large compartments, which supports our unbiased CPP paradigm. This paradigm took place in five consecutive days, which consisted of three distinct phases: preconditioning, conditioning, and postconditioning. In all phases, the animals were tested during the same time period each day.

Preconditioning phase. On day 1 (pre-exposure), each rat with free access to all compartments was placed separately in the apparatus for 10 minutes. Animal displacement was recorded and analyzed on this day (pretest day). In the experimental setup used in this study, the animals did not show an unconditioned preference for any compartment. Animals were then randomly assigned to one of the groups for place conditioning and 6–8 animals were used in each following experiment.

Conditioning phase. This phase consisted of a 3-day plan of conditioning sessions. In this phase, the animals received two trials in which they experienced the effects of the drugs while enclosed to one compartment for 30 min and other trials in which they experienced the effects of saline while enclosed in the other compartment by closing the removable wall. Access to the compartments was blocked on these days.

Postconditioning phase. On the fifth day (test day), the removable wall was removed, and the rats could access the entire apparatus. The mean time spent in compartments during a 10-min period was recorded for each rat. In order to calculate the conditioning score, the difference in time spent for the drug-paired place and saline-paired place (two equal size compartments) was measured as the preference criteria. Time spent in each compartment and animal displacement were recorded by using a camera (Panasonic) placed 2 m above the CPP boxes and locomotion tracking was measured by Maze router software (Science Beam company, Iran). A video tracking system for automation of behavioral experiments was used (Ebrahimian, Naghavi, Yazdi, Sadeghzadeh, Taslimi, & Haghparast, 2016).

### 2.4. Induction of METH extinction

Following the preference test day, the animals during the conditioning phase were exposed to extinction training with access to all compartments in the CPP apparatus without any drug injection for 30 min each day. This procedure was repeated for each animal in the control and experimental groups until the calculated CPP scores in two consecutive days in extinction period became similar to those in the preconditioning day. Conditioning score (CPP score) represents the time spent in the drug-paired place minus the time spent in saline-paired place which were recorded by Maze router software. Thus, the criterion for extinction or maintenance of the METH rewarding properties in all groups was the lack of significant differences in preference scores between two consecutive days in the extinction period and the preference score on the preconditioning day (Haghparast, Omranifard, Arezoomandan, Ghalandari-Shamami, Taslimi, Vafaei, 2013).

### 2.5. Restraint Stress Test

To induce acute stress induction, the animals were immobilized for 3 h once a day just before the reinstatement phase. For chronic stress, the rats were exposed to immobilization stress for 1 h daily during extinction period in rodent immobilization bags (Santibanez, Gysling, & Forray, 2006; Vyas, Bernal, & Chattarji, 2003). Briefly, each rat was placed in an acrylic mesh restrainer device (length 20 cm, width 7 cm, height 6 cm) while control rats were kept in their home cages. Immediately after that, all animals were tested for reinstatement of METH-CPP (Quadros & Miczek, 2009).

### 2.6. Experimental design

#### 2.6.1. METH dose-response effect on conditioned place preference paradigm

In these experiments, a dose–response relationship for METH on CPP paradigm was established. Different doses of METH (0.125, 0.25, 0.5, 1, 2 and 4 mg/kg) were injected subcutaneously, to CPP induction during three days of conditioning phase (acquisition). In the control group, the animals received saline instead of METH.

#### 2.6.2. Reinstatement of extinguished METH-induced condition place preference in rats

**Figure 1:**
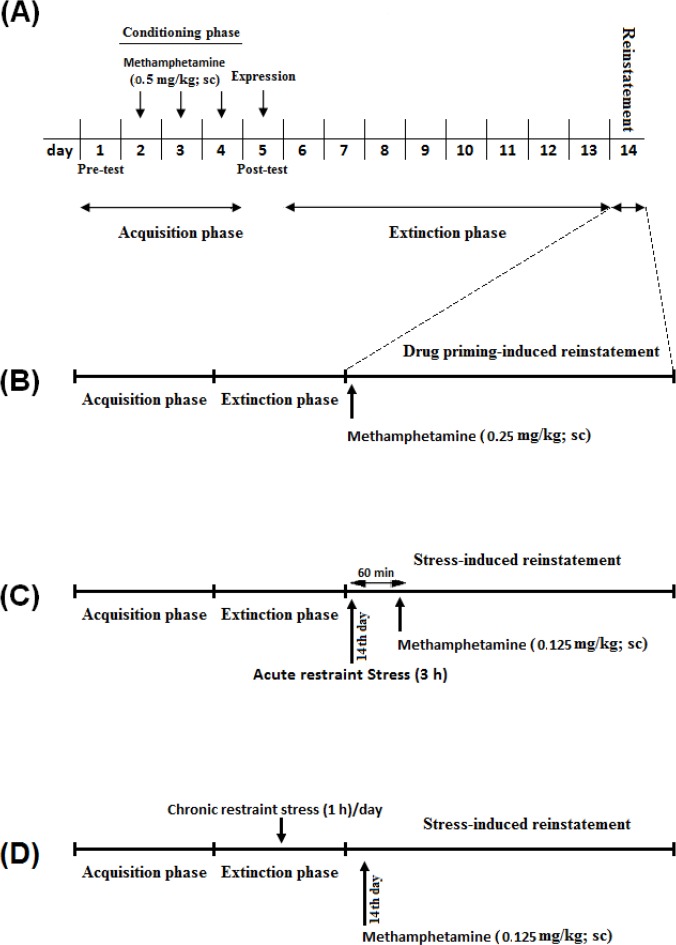
A) Experimental protocols for METH-induced reinstatement of conditioned place preference (CPP) in the rats. B) In the set of experiments, 24 hours after the last day of the extinction period, the animals were placed in the CPP box and tested for CPP test by only injection of the priming dose of METH (0.25 mg/kg; SC). C) In the set of experiments (ineffective METH dose + acute restraint stress-induced reinstatement), after extinction was established, the rats were given a 3-h restraint stress period and after 60 min, they were placed in the CPP box and tested for reinstatement by only injection of the ineffective dose of METH (0.125 mg/kg; SC). D) In the set of experiments, after the CPP acquisition, 60 min before animals placing into the CPP box, they received 1 hour restraint stress as a chronic stress period every day in the extinction period, 24 hours after the last day of the extinction period. The animals were tested for reinstatement by only injection of the ineffective dose of METH (0.125 mg/kg; SC).

In this set of experiments, the animals were exposed during three days to one distinct chamber in the presence of METH (0.5 mg/kg; SC) and alternative chamber in the presence of vehicle (Saline). The day after the test day, the animals were given free access to both chambers for 8 days (extinction phase). To assess the METH-induced reinstatement, two groups of animals treated with ineffective dose of METH (0.125 and 0.25 mg/kg; SC), and another group received saline as a vehicle group. Conditioning score and distance traveled were recorded during reinstatement phase during a 10-min period (Figure 1 A, B).

#### 2.6.3. Effect of exposure to acute restraint stress on reinstatement of METH-induced CPP in rats

To examine the possible role of acute application of restraint stress on reinstatement of extinguished METH-induced CPP, animals passed conditioning and extinction phase. The day after extinction, the rats were exposed to the restraint stress for 3-h period, and after 60 min, were placed in CPP apparatus and received ineffective dose of METH (0.125 mg/kg) to induce reinstatement phase. Conditioning score and distance traveled were recorded during 10-min period (Figure 1 C).

#### 2.6.4. Effect of exposure to chronic restraint stress on reinstatement of METH-induced CPP in rats

In order to examine the possible effect of chronic restraint stress in reinstatement of extinguished METH-induced CPP, after conditioning phase, the animals received restraint stress for 1 h every day. Sixty minutes later, the animals were placed in entire apparatus (free access) to induce extinction phase. The day after extinction phase, the animals received ineffective dose of METH (0.125 mg/kg) to induce reinstatement of METH (Figure 1 D).

### 2.7. Statistics

Conditioning score represents the difference between the times spent in the drug- and saline-paired compartments, and is expressed as mean ± SEM (standard error of mean). Data were processed by commercially available software Graph Pad Prism® 5.0. In order to compare the conditioning scores and distance traveled obtained in all groups (vehicle and experimental groups), 1-way analysis of variance (ANOVA) and repeated measures or randomized block model followed by post hoc analysis (Dunnett’s or Newman-Keuls test) were used as appropriated. P-values less than 0.05 were considered to be statistically significant.

## 3. Results

**Figure 2:**
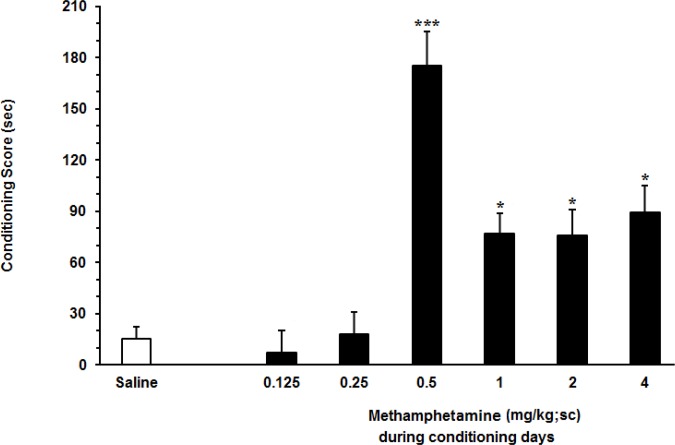
Effect of different doses of METH on place preference in rats Each point shows the Mean±SEM for 7–10 rats in each group; * P<0.05 and *** P<0.001 compared with saline-control group.

In the first set of experiments, we examined the dose response effects of different doses of METH (0.125, 0.25, 0.5, 1, 2 and 4 mg/kg) injected subcutaneously, on CPP paradigm (n=8). One-way ANOVA followed by Dunnett’s test (F6, 55=17.25, P<0.0001) revealed significant differences in conditioning scores among the vehicle (saline) and experimental groups (Figure 2). Our findings showed that the most effective dose of METH is 0.5 mg/kg (P<0.001).

### 3.1. Reinstatement of extinguished METH induced CPP in rats

**Figure 3:**
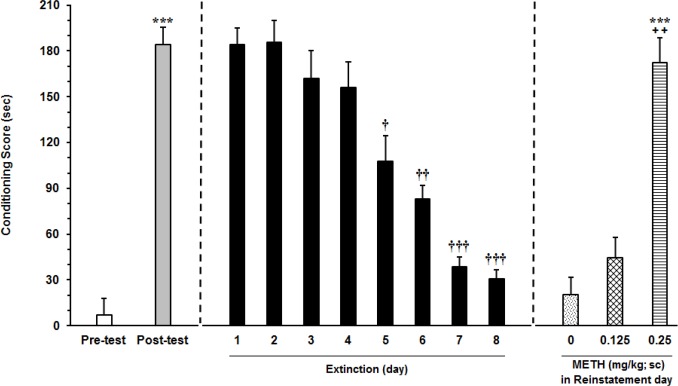
Dose–response effects of METH on the reinstating of extinguished METH-induced conditioned place preference In the right panel, animals received METH (0.5 mg/kg; SC) during conditioning phase, tp (5)=8.598 (P<0.001). The day after postconditioning day, the animals were given free accesses to both chambers for 8 days. To assess the METH-induced reinstatement, the animals received ineffective doses of METH, i.e., 0.125 and 0. 25 mg/kg and saline as a control group. Each column represents the Mean±SEM parameter values of 6 rats; *** P<0.001 compared with the pretest day; † P<0.01, †† P<0.001 and ††† P<0.0001 different from the posttest day; ++ P<0.01 compared with the last day of extinction period.

Subcutaneous injection of METH (0.5 mg/kg) during 3 conditioning days induced significant preference (P<0.001) for the METH-paired chamber in comparison with saline-paired chamber. During the extinction period, without any injection, the CPP score was calculated every day. The CPP induction by METH was gradually moderated over days and the time spent in METH-paired chamber did not differ from the saline one by the seventh and eighth extinction day. After the last extinction day, the animals were tested for reinstatement. Subcutaneous injection of METH priming dose (0.25 mg/kg) could induce reinstatement, (F_3,23_=8.031, P<0.0001) and CPP score on the reinstatement day significantly increased compared to pretest phase (P<0.001) (Figure 3) (n=8).

### 3.2. Effect of exposure to acute restraint stress on reinstatement of METH-induced CPP in rats

**Figure 4:**
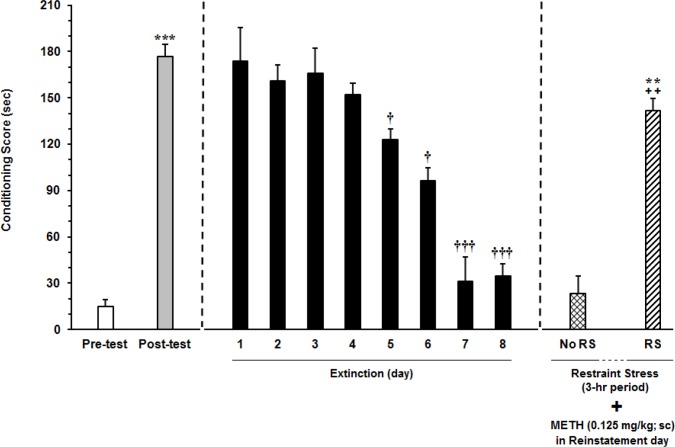
Effect of exposure to acute restraint stress on the reinstatement of METH-induced CPP in rats The day after extinction, the animals were faced with acute restraint stress (3-h period). The animals received ineffective dose of METH for reinstatement (0.125 mg/kg). In reinstatement phase, conditioning scores were recorded during 10-min period. Each column represents the mean ± SEM parameter values of 7 rats; ** P<0.01 and *** P<0.001 compared with the pretest day; † P<0.01 and ††† P<0.0001 different from CPP test in the postconditioning phase; ++ P<0.01 compared with the last day of extinction period.

In this set of experiments, the possible effect of acute restraint stress on reinstatement of extinguished METH-induced CPP was examined. Animals passed conditioning and extinction phase as described before but the day after extinction (reinstatement phase), animals were exposed to restraint stress for 3-h period, (F_8,62_=6.644, P<0.0001) (Figure 4). Animals received ineffective dose of METH for reinstatement (0.125 mg/kg), after exposure to acute restraint stress. Conditioning score and distance traveled were recorded during 10-min period (F_2,20_=12.27, P=0.0004) (n= 8). As results shown, ineffective dose of METH for reinstatement induction, together with acute restraint stress could result in METH reinstatement.

### 3.3. Effect of exposure to chronic restraint stress during extinction phase on the reinstatement of METH-induced CPP in rats

**Figure 5:**
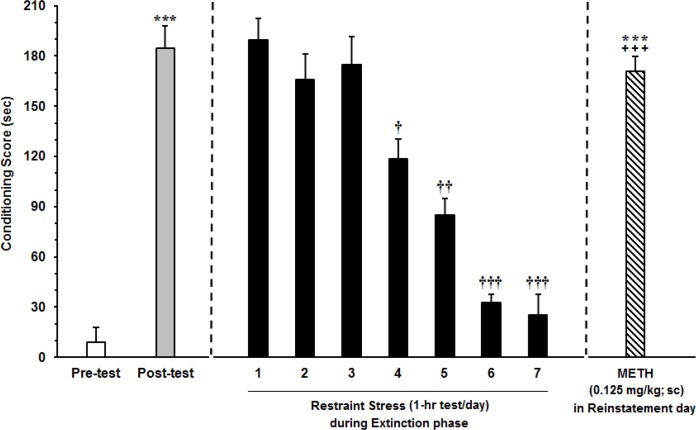
Effect of exposure to chronic restraint stress on reinstatement of METH-induced CPP in rats The animals were exposed to restraint stress every day during extinction phase 1 hour per day. After relapse, the animals received ineffective dose of METH for reinstatement (0.125 mg/kg). Each column represents the Mean±SEM parameters values of 8 rats; *** P<0.001 compared with the pretest day; † P<0.01, †† P<0.001 and ††† P<0.0001 different from the posttest day; +++ P<0.001 compared with the last day of extinction period.

To assess the chronic stress effects on reinstatement of METH before putting the animals in CPP apparatus, they were exposed to the restraint stress for 1 hour every day during the extinction phase. The CPP score was calculated every day (Figure 1). In this experiment, group chronic stress could diminish extinction phase for one day (F_7,63_=7.998, P<0.0001) (Figure 5) (n=8). The day after extinction phase, the animals in chronic stress group received ineffective dose of METH for reinstatement (0.125 mg/kg). Comparing the conditioning score between reinstatement day and pretest using student t test showed significant difference (t_p_[7]=6.271, P<0.001) (Figure 5) indicating that METH ineffective dose for reinstatement induction, together with chronic restraint stress, could result in reinstatement of METH.

## 4. Discussion

Stressful situations modify functions in areas of the brain involved in the rewarding effects of psychostimulants. Although all factors responsible for relapse to drug seeking are not completely known, addicting drugs and stress are considered to bring about medication craving and reinstatement of extinguished drug-seeking in retrieving drug abusers (Sadeghzadeh, Babapour, & Haghparast, 2016). The major finding of our study was that the acute and chronic restraint stress potentiates the effect of low-dose METH and could reinstate METH conditioning place preference in the rats. Nevertheless, for the first time, our data provided evidence that chronic immobilization stress could reduce duration of extinction of METH-induced conditioning place preference. Thus the results of the current research further add to the growing literature on the association of stress with urge and reward pathway.

Drug-associated stimuli, stress, and drugs of abuse are hypothesized to trigger reinstatement to drug reward-related behaviors (Lu, Shepard, Hall, & Shaham, 2003). Previous studies have shown that stressors, such as restraint (Pacchioni, Gioino, Assis, & Cancela, 2002), footshock (Wang, Luo, Ge, Fu, & Han, 2002), butt pinching (Katz & Roth, 1979), and defeat (Covington & Miczek, 2001), efficiently cause drug reward and reinstatement. Interestingly, this current analysis also proves that the duration of restraint stress for causing reinstatement of extinguished METH-CPP. This is despite the fact that acute and chronic stressors have a different and separate efficiency.

It has already been shown that acute restraint stress activates orexin neurons in the lateral hypothalamus, which send projections to the ventral tegmental area, releasing orexins that activate dopaminergic neurons and reward pathway (Tung et al., 2016). Several lines of evidence have also suggested that the reinstatement of drug seeking behaviors is mediated by dopamine receptors (Dai, Kang, Wang, & Ma, 2006; Gilbert et al., 2005). Likewise, Mazid et al. suggested that desperate stress could affect opioid-related learning (Mazid, Hall, Odell, Stafford, Dyer, Van Kempen, et al., 2016). Thus, it confirms that desperate food deprivation facilitate reinstatement of morphine CPP in rats (Sadeghzadeh et al., 2016). Furthermore acute social defeat stress involve on the reinstatement of the CPP caused by cocaine (Montagud-Romero, Aguilar, Maldonado, Manzanedo, Minarro, & Rodriguez-Arias, 2015).

Conrad et al. reported that cold swim stress can have long-term results on cocaine seeking habit (Conrad et al., 2010). On the other hand, experiments show that chronic stress significantly decreases cocaine-induced activation of reward pathway (Glynn et al., 2016). Also, it has been reported that exposure to chronic stress protocol significantly reduces dopamine extracellular levels induced by cocaine (Sotomayor-Zarate, Abarca, Araya, Renard, Andres, & Gysling, 2015). Past studies have shown that repeated restraint stress with direct exposure enhances excitatory drive to the basolateral amygdala, an area critical for behavioral responses to be anxious (Padival, Quinette, & Rosenkranz, 2013). As we mentioned, chronic stress in our study decreased duration of METH extinction, and animal exposure to reinstatement phase, sooner than those received acute stress or the control group. It seems that chronic stress affects the reward pathway and extinction process since chronic stress may have an aversive impact on daily living, so individuals may tend to cope using drugs. Chronic stress has worst effects than desperate stress. Exposure to stress prevails in all life events and hypothalamic-pituitary-adrenal (HPA) axis dysfunction has been implicated in the development of several psychological disorders that are comorbid with craving (Faravelli et al., 2012).

Mahoney et al. mentioned that stimulant users endorse greater impulsivity, life stress and sensation seeking; however, methamphetamine users endorsed significantly higher number of life stressors and increased life stressors may account for their methamphetamine usage patterns (Mahoney, Thompson-Lake, Cooper, Verrico, Newton, & De La Garza, 2015). Backing the value of environment in drug addiction, our data support the idea that the restraint stress evokes the reinstatement of METH-CPP responses. Together, these studies raise the intriguing opportunity that the behavioral impact of stress exposure on incubation of reinstatement could also be due to alterations in activity within the brain area involved in stress process.

In summary, acute and chronic restraint stress could reinstate METH CPP by ineffective dose of METH for reinstatement induction. These studies will finally guide us to develop effective ways to cut down craving and prevent urge in abstinent amphetamine abusers. However, further behavioral, electrophysiological and molecular investigations are needed to elucidate brain areas involved in psychological stress and medication relapse.

## Ethical Considerations

### Compliance with ethical guideline

Each animal was used only once. Rats got familiar with their new environment prior to starting experimental process. All tests were executed according to the guide for the care and use of laboratory animals (National Institutes of Health Newsletter No. 80-23, revised 1996). The study was approved by the Research and Ethics Committee of Hamadan University of Medical Sciences, Hamadan, Iran.
